# Case Report and Preliminary Exploration: Protection of Supraclavicular Nerve Branches during Internal Fixation of Clavicular Fractures through Preoperative Ultrasound Localization

**DOI:** 10.3389/fsurg.2022.898664

**Published:** 2022-05-27

**Authors:** Yulin Wang, Jiapeng Huang, Jianjun Li, Jinfeng Zhou, Qiang Zheng, Zhixue Chen, Penghui Wei, Wenxi Tang

**Affiliations:** ^1^ Department of Anesthesiology, Qilu Hospital (Qingdao), Cheeloo College of Medicine, Shandong University, Qingdao, China; ^2^ Department of Anesthesiology and Perioperative Medicine (JH), University of Louisville, Louisville, KY, United States of America; ^3^ Department of Orthopedics and traumatology, Qilu Hospital (Qingdao), Cheeloo College of Medicine, Shandong University, Qingdao, China

**Keywords:** ultrasound, supraclavicular nerve, clavicle fracture, internal fixation, neuroprotection, case report

## Abstract

**Introduction:**

Protecting the supraclavicular nerve during internal fixation of clavicular fractures can reduce numbness in its innervation area after surgery. Previous methods for supraclavicular nerve protection are empirical, time-consuming, and approximate. In this report, we verified the feasibility of using ultrasound for percutaneous localization of the terminal branches of the supraclavicular nerve and the feasibility of an ultrasound-guided skin flap reserve technique for nerve protection.

**Case Presentations:**

A high-frequency linear array probe was used in three cases to trace the supraclavicular nerve from its origin at the superficial cervical plexus on the surface of the clavicle. In the first case, the feasibility of percutaneous ultrasound localization of the terminal branches of the supraclavicular nerve was determined by performing an ultrasound-guided nerve block. In the second case, the feasibility of this method was determined by directly isolating this nerve under direct vision. In the third case, after the ultrasound localization, the nerves were protected by intraoperative skin retention. In the first case, skin anesthesia of the innervation area of the intermediate branch of the supraclavicular nerve was achieved. In the second case, the part of the nerve that crosses the surface of the clavicle was quickly found and successfully protected, and no obvious abnormal skin sensations were noted after the operation. In the third case, there was no abnormal sensation in most of the associated skin except for the innervation area of the lateral branch of the supraclavicular nerve.

**Conclusions:**

The medial and intermediate branches of the supraclavicular nerve could be located over the skin by ultrasound, and this could be helpful in quickly isolating these nerves intraoperatively. Retaining the corresponding skin can protect the function of these nerve branches and effectively reduce the area of skin numbness after surgery.

## Introduction

Fractures of the clavicle are frequent injuries and account for 2%–5% of all fractures of the human body (1, 2), and nearly 80% of clavicle fractures occur in the middle segment (3). Steel plates are considered the gold-standard treatment for midclavicular fractures (4). The branches of the supraclavicular nerve are the main sensory nerves distributed in the skin over the clavicle and its surroundings. Conventional open reduction with internal plate fixation of clavicular fractures can easily damage the branches of the supraclavicular nerve, and anterior chest wall numbness secondary to injury of these branches is a common complication of this operation. The reported incidence of this complication is 55.3%–86%, and it is a burden to 61.9% of these patients. Various degrees of cutaneous numbness are reported to persist in 66.7% of patients and may become permanent (5–7). Protection of the supraclavicular nerve during internal fixation of clavicular fractures could significantly reduce the numbness and other postoperative discomforts (5,8–10).

Oblique incisions, mini-open plating, and preservation of the supraclavicular nerve under direct vision are common approaches to ensure neuroprotection (8, 9). Neuroprotection achieved through an oblique incision and mini-open plating depends on the surgeon’s experience and is time-consuming (5). All these methods carry the potential risk of unintentional nerve injury. The mean diameters of the common trunk, intermediate branch, and lateral branch of the supraclavicular nerve are 4.1, 2.5, and 2.7 mm, respectively (11). Ultrasound-guided blocks of these nerve branches have been used by anesthesiologists. However, there are no reports describing the diameters of the terminal branches of the supraclavicular nerve (over and adjacent to the clavicle) and how to identify them with ultrasound. We hypothesized that the terminal branches of the supraclavicular nerve can be located through the skin by ultrasound and that this could aid in protecting the supraclavicular nerve during internal fixation of clavicular fractures. We verified this hypothesis using three cases in this study. This study was approved by the Medical Ethics Committee of the Qilu Hospital (Qingdao), Shandong University. Written informed consent was obtained from all the patients.

## Case Presentation

### Case 1

In the first case, we tested whether the terminal branches of the supraclavicular nerve could be traced out by ultrasound, as no previous studies had shown their ultrasound images.

**Figure 1:**
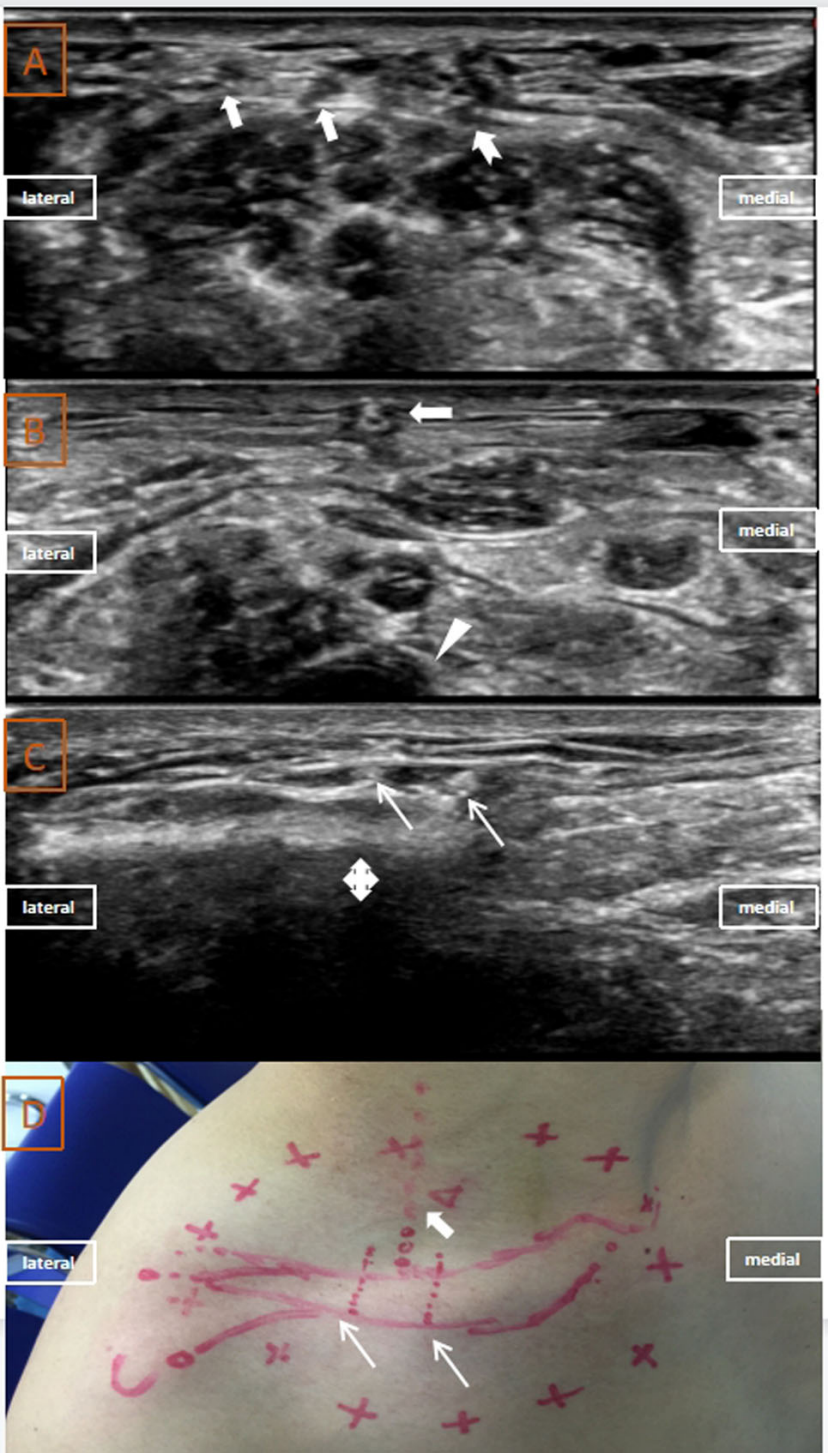
Ultrasound images, projection on the body surface and sensory block area of the supraclavicular nerve in Case 1: (**A**) ultrasound images of phrenic nerve (indicated with swallowtail arrow) and superficial cervical plexus (indicated with a thick white arrow); (**B**) ultrasound image of the supraclavicular nerve (indicated with a thick white arrow) anterior to the supraclavicular artery (indicated with a white arrowhead); (**C**) ultrasound images of branches of the supraclavicular nerve (indicated with thin white arrows) anterior to the clavicle (indicated with a cross arrow); (**D**) projection of the supraclavicular nerve on the body surface (indicated with a white arrow) and the sensory block area (red circle).

The first case was of a 37-year-old healthy man (the first author of this article). A 15 MHZ high-frequency linear array probe (Wisonic Medical Technology, Shenzhen, China) was placed at the mid-posterior border of the sternocleidomastoid muscle (SCM). Four hypoechoic dots were identified on the surfaces of the anterior and medial scalene muscles. One dot was found on the surface of the scalenus anterior muscle (the phrenic nerve), and the other three dots were on the surface of the medial scalenus muscle; these were believed to be branches of the superficial cervical plexus (Figure 1A). The probe was then moved caudally, and one of the branches of the superficial cervical plexus extended down to the clavicle and anterior to the supraclavicular artery, which is an oval hypoechoic structure (Figure 1B). Approximately 1 cm closer to the superior margin of the clavicle, this image disappeared, and two fascicular hyperechoic masses emerged from the hypodermal tissue and extended to the clavicle (Figure 1C). These two hyperechoic structures were believed to be the main branches of the supraclavicular nerve. Anterior to the supraclavicular artery, 2% lidocaine (0.5 ml) was injected near these nerves using the out-of-plane technique. Sensory blockade was evaluated after 5 min (Figure 1D), and the evaluation confirmed that the medial and intermediate rami of the supraclavicular nerve were located correctly. The path of the supraclavicular nerve was therefore traced and marked on the skin under ultrasound guidance (Figure 1D).

### Case 2

In the second case, we confirmed the feasibility of locating the terminal branches of the supraclavicular nerve by separating the nerve under direct vision (separating the nerve under direct vision is a common neuroprotection technique).

**Figure 2:**
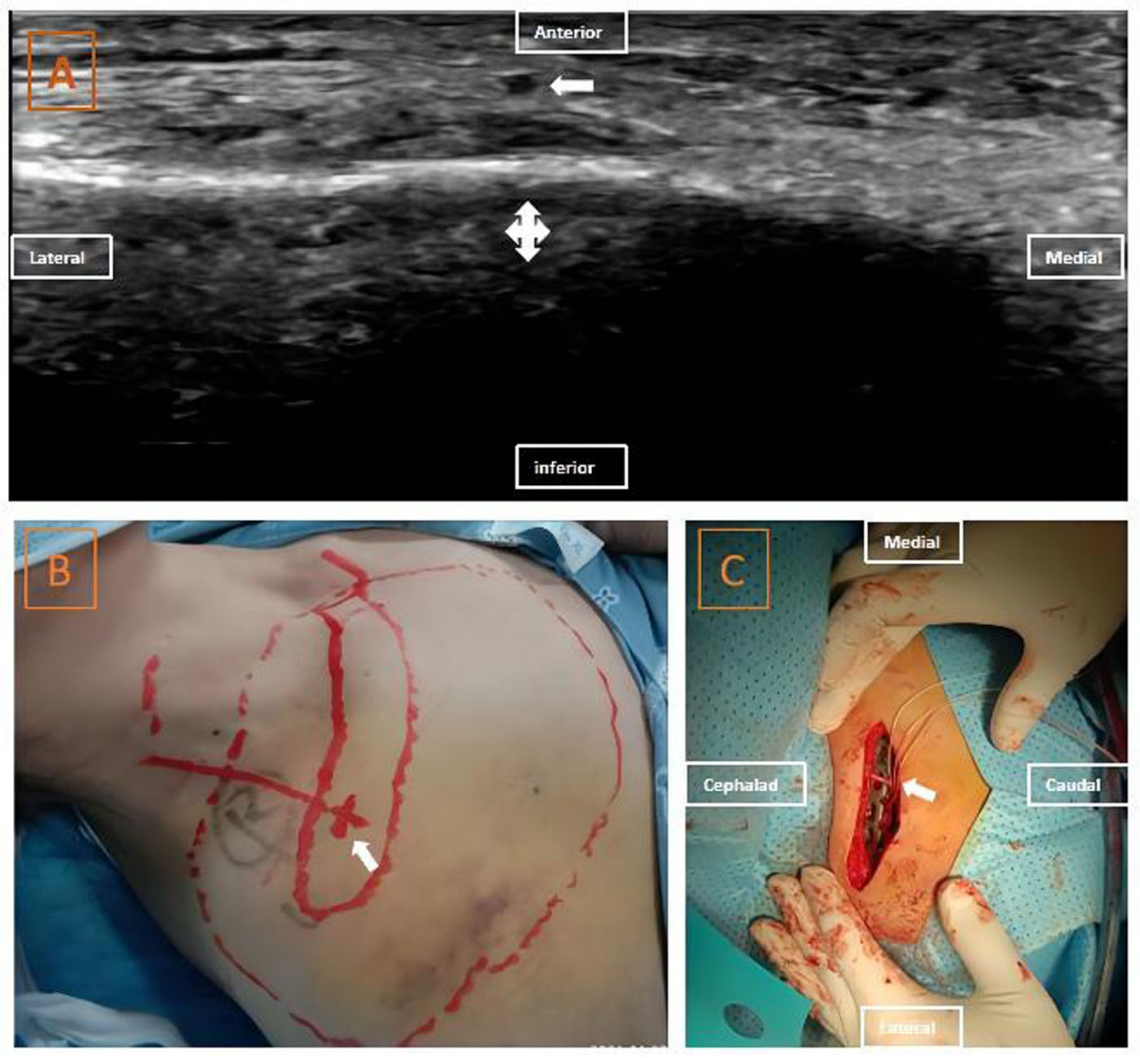
Ultrasound images, projection on the body surface, sensory block area and intraoperative view of supraclavicular nerve in case 2: (**A**) ultrasound images of the supraclavicular nerve anterior to the clavicle; (**B**) projection of the supraclavicular nerve on the body surface and the sensory block area. (**C**) intraoperative view of the supraclavicular nerve. The thick white arrow indicates the supraclavicular nerve; the cross arrow indicates the clavicle.

Case 2 involved a 69-year-old woman who was scheduled for an open reduction and internal fixation of a right clavicle fracture. Two milligrams of midazolam and 50 µg of fentanyl intravenous injection were given to sedate the patient and alleviate her pain. Using our previous method, we identified the possible supraclavicular nerve by tracing it caudally from the superficial cervical plexus to where it crosses the clavicle (Figure 2A). About 0.5 ml of 2% lidocaine was injected near the nerve using the out-of-plane technique. Sensory blockade was evaluated as previously described (Figure 2B), and it proved that these suspected nerves were the medial and intermediate rami of the supraclavicular nerve. Thence, 1 min after skin incision, this nerve was found in the subcutaneous tissue near the puncture point of the supraclavicular nerve block. Under direct visualization, the nerve was located over the middle of the clavicle, and it extended beyond it (Figure 1C). It was carefully separated from the clavicle and protected using rubber strips. A steel plate was placed under the supraclavicular nerve to prevent nerve injury (Figure 1C). Follow-up at 24 h after surgery revealed that the patient had no obvious numbness in the skin areas around the clavicle and no significant sensory deficit. In this case, we did not find the inferior branch of the intermediate ramus of the supraclavicular nerve neither with ultrasound nor with open direct visualization; this may be attributed to anatomical variation.

### Case 3

Finally, in case 3, we verified the feasibility of protecting the supraclavicular nerve by retaining the skin flap over it during surgery (this is an innovative technique proposed by our team).

**Figure 3:**
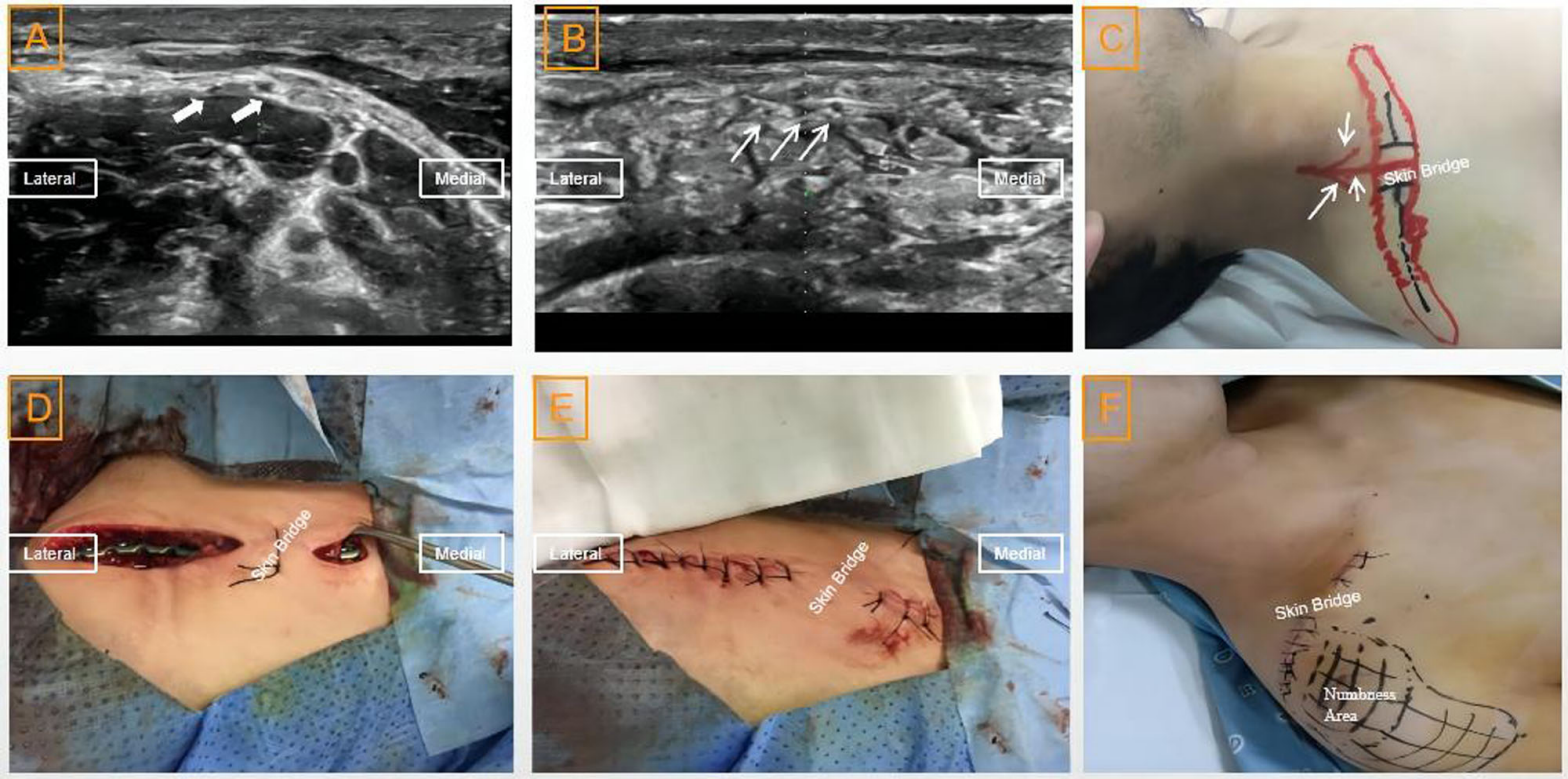
Ultrasound images, projection on body surface of the supraclavicular nerve, skin flap (bridge) and numbness area after the operation in case 3: (**A**) ultrasound images of the branches of the supraclavicular nerve at its origin (indicated with a thick white arrow); (**B**) ultrasound images of the branches of the supraclavicular nerve (indicated with a thin white arrow) anterior to the clavicle (indicated with a white cross arrow); (**C**) projection of the branches of the supraclavicular nerve on the body surface and skin flap (bridge) before the operation; (**D**) skin flap during the operation; (**E**) skin flap after the operation; and (**F**) numbness area after the operation.

This case was of a 27-year-old man scheduled for an internal plate fixation indicated for a right clavicle fracture. Similarly, after intravenous injection of the sedative and analgesic, we used the previously described method to identify the three possible branches of the supraclavicular nerve at the surface of the clavicle (Figures 3A, B). The paths of these nerves were marked on the skin, and the incision line was designed to avoid damaging these nerves (Figure 3C). We made a horizontal incision, retaining a skin bridge (flap) over the nerve in the middle of the clavicle, and cutting the skin from both ends of the clavicle (Figures 3D, E). The steel plate was then placed over the periosteum of the clavicle through a subcutaneous tunnel (separation of the subcutaneous tissue and insertion of the plate were carefully performed tightly against the periosteum to minimize nerve injuries) (Figure 3D). On the third postoperative day, the patient complained of numbness and hyperalgesia in the skin below the lateral one-third of the clavicle. The sensation in the areas below the medial two-thirds of the clavicle was normal (Figure 3F).

## Discussion

Our report suggests that locating the important distal branches of the supraclavicular nerve with ultrasound is possible and protecting these nerves by retaining the skin over them is feasible. However, sensory loss of the lateral one-third area was still present in the third case, and this may be due to the transection of the lateral branch of the supraclavicular nerve.

The supraclavicular nerve is a cutaneous sensory nerve originating from the C3 and C4 roots of the superficial cervical plexus. The supraclavicular nerve commonly divides into intermediate and lateral branches, and in some cases, there may be an additional medial branch (11–13). In most cases, these branches arise from a common trunk behind the posterior border of the SCM. The intermediate ramus emerges beneath the posterior border of the SCM, descends distally and anteriorly, and divides into two or three secondary branches. Its most medial branch crosses the middle third of the clavicle and its most lateral branch crosses the second lateral quarter of the clavicle to supply sensory innervations to the skin over the anterior upper part of the chest. The lateral ramus passes directly towards the acromial process, crosses the anterior border of the trapezius muscle, and divides into several branches that supply the skin of the shoulder. The medial ramus runs down along the posterior edge of the SCM to the clavicle, and then crosses through the platysma to provide sensory innervation to the sternal notch (11–13).

Open reduction and internal fixation of clavicular fractures can easily cause iatrogenic injury to branches of the supraclavicular nerve, and in the absence of neuroprotective measures, more than half of the patients develop numbness and other discomforts that affect their quality of life (5–7). Supraclavicular nerve-sparing techniques can reduce the incidence of anterior chest wall numbness from 86% to 35% (5). Therefore, surgeons advocate for the protection of the supraclavicular nerve during surgery. Previous methods of protecting the supraclavicular nerve include oblique incision, mini-open plating, and direct preservation of the nerve after horizontal skin incisions. The first two techniques are unreliable because they are based on the surgeon’s experience in determining the location of the nerves. The latter technique requires a longer surgical time to dissect the nerve. Preoperative ultrasound localization might help surgeons to easily locate the nerve (before incision) and adjust the incision away from the nerves (e.g., preservation of the skin flap over the supraclavicular nerve after ultrasound localization).

Selective supraclavicular nerve block guided by ultrasound has been reported in some surgeries (14, 15). However, to the best of our knowledge, there are no studies on this technique for intraoperative neuroprotection, and no studies have reported the technique of identifying terminal branches of the supraclavicular nerve using ultrasound images taken over the surface of the clavicle.

Ultrasound and magnetic resonance imaging (MRI) are imaging methods that can be used to identify nerves; however, the use of MRI has not been reported for cutaneous nerve identification (16). For nerves, the resolution of ultrasonic techniques is stronger than that of MRI (17, 18). In addition, ultrasound is less expensive and more convenient. Ultrasound can be used to continuously track down a nerve along its path while marking the corresponding location on the overlying skin surface; this is a unique advantage of ultrasonic techniques over MRI.

This is our preliminary study on the use of ultrasound imaging to protect supraclavicular nerve branches during surgery. We acknowledge the following limitations of this study. First, according to anatomy (11–13), the innervation area of the lateral branch of the supraclavicular nerve did not appear to be affected by the nerve blocks in cases 1 and 2, whereas case 3 showed injury of the lateral branch of the supraclavicular nerve. This indicates that we failed to locate the terminal rami of the lateral branch of the supraclavicular nerve. This may be due to the smaller diameter of these rami, low ultrasound image contrast with the surrounding tissue, or poor resolution of the equipment. The use of a higher frequency myoskeletal ultrasound-dedicated probes may help identify all branches of the supraclavicular nerve. Second, because the identification of these nerves partially depends on the surgeon’s mastery of anatomy, this newly introduced technique of preoperative localization of the supraclavicular nerve is limited by anatomical variations of the nerve and its branches.

In conclusion, preoperative ultrasound localization of the terminal rami of the medial and intermediate branches of the supraclavicular nerve was feasible and could help avoid iatrogenic nerve injury during surgery.

## Data Availability

The original contributions presented in the study are included in the article/supplementary material; further inquiries can be directed to the corresponding author.
